# In-Home Particle Concentrations and Childhood Asthma Morbidity

**DOI:** 10.1289/ehp.11770

**Published:** 2008-10-24

**Authors:** Meredith C. McCormack, Patrick N. Breysse, Elizabeth C. Matsui, Nadia N. Hansel, D’Ann Williams, Jean Curtin-Brosnan, Peyton Eggleston, Gregory B. Diette, for the Center for Childhood Asthma in the Urban Environment

**Affiliations:** 1 Department of Medicine, Johns Hopkins University School of Medicine, Baltimore, Maryland, USA; 2 Department of Environmental Health Sciences, Johns Hopkins Bloomberg School of Public Health, Baltimore, Maryland, USA; 3 Department of Pediatrics, Johns Hopkins University School of Medicine, Baltimore, Maryland, USA; 4 Department of Epidemiology, Johns Hopkins Bloomberg School of Public Health, Baltimore, Maryland, USA

**Keywords:** air pollution, asthma, indoor, particulate matter, pediatric, urban

## Abstract

**Background:**

Although outdoor particulate matter (PM) has been linked to mortality and asthma morbidity, the impact of indoor PM on asthma has not been well established.

**Objective:**

This study was designed to investigate the effect of in-home PM on asthma morbidity.

**Methods:**

For a cohort of 150 asthmatic children (2–6 years of age) from Baltimore, Maryland, a technician deployed environmental monitoring equipment in the children’s bedrooms for 3-day intervals at baseline and at 3 and 6 months. Caregivers completed questionnaires and daily diaries during air sampling. Longitudinal data analyses included regression models with generalized estimating equations.

**Results:**

Children were primarily African Americans (91%) from lower socioeconomic backgrounds and spent most of their time in the home. Mean (± SD) indoor PM_2.5–10_ (PM with aerodynamic diameter 2.5–10 μm) and PM_2.5_ (aerodynamic diameter < 2.5 μm) concentrations were 17.4 ± 21.0 and 40.3 ± 35.4 μg/m^3^. In adjusted models, 10-μg/m^3^ increases in indoor PM_2.5–10_ and PM_2.5_ were associated with increased incidences of asthma symptoms: 6% [95% confidence interval (CI), 1 to 12%] and 3% (95% CI, –1 to 7%), respectively; symptoms causing children to slow down: 8% (95% CI, 2 to 14%) and 4% (95% CI, 0 to 9%), respectively; nocturnal symptoms: 8% (95% CI, 1 to 14%) and 6% (95% CI, 1 to 10%), respectively; wheezing that limited speech: 11% (95% CI, 3 to 19%) and 7% (95% CI, 0 to 14%), respectively; and use of rescue medication: 6% (95% CI, 1 to 10%) and 4% (95% CI, 1 to 8%), respectively. Increases of 10 μg/m^3^ in indoor and ambient PM_2.5_ were associated with 7% (95% CI, 2 to 11%) and 26% (95% CI, 1 to 52%) increases in exercise-related symptoms, respectively.

**Conclusions:**

Among preschool asthmatic children in Baltimore, increases in in-home PM_2.5–10_ and PM_2.5_ were associated with respiratory symptoms and rescue medication use. Increases in in-home and ambient PM_2.5_ were associated with exercise-related symptoms. Although reducing PM outdoors may decrease asthma morbidity, reducing PM indoors, especially in homes of inner-city children, may lead to improved asthma health.

Particulate matter (PM) is one of six damaging air pollutants that has been identified under the Clean Air Act of 1970 and is regulated for the sake of protecting human health. The harmful effects of outdoor PM have been well established and include premature death (Samet et al. 2000) and worsening of asthma morbidity (Mar et al. 2004; Pope et al. 1991; Rabinovitch et al. 2006; Romieu et al. 1996; Yu et al. 2000). The evidence of the effect of indoor PM on asthma health is not as well established. The indoor environment is especially important in the context of asthma because Americans spend most of their time indoors, and improvement of indoor air quality could therefore provide an important opportunity to better asthma health. Indoor PM differs from outdoor PM in concentration, source, and composition (Allen et al. 2003; Long et al. 2000; Turpin et al. 2007; Wallace 1996; Wallace et al. 2003). Thus, we cannot simply extrapolate the health effects of indoor PM from studies of outdoor air.

Under the Clean Air Act of 1970, the National Ambient Air Quality Standards (NAAQS) have defined limits for acceptable daily and annual concentrations of outdoor PM < 2.5 μm and < 10 μm in diameter (PM_2.5_ and PM_10_, respectively) [U.S. Environmental Protection Agency (EPA) 1997]. More recently, there has been interest in developing standards for the coarse thoracic particles that are between 2.5 and 10 μm in diameter (PM_2.5–10_), which, unlike PM_10_, do not include the PM_2.5_ (fine PM) fraction. In the recent 2006 NAAQS update, PM_2.5_ standards were made more stringent based on substantial scientific evidence of detrimental health effects (U.S. EPA 2006b). There was a simultaneous proposal to replace the existing daily PM_10_ standards with a PM_2.5–10_ (coarse PM) standard in urban areas only (U.S. EPA 2006b, 2006c). However, in the final NAAQS, the annual PM_10_ standard was revoked and the daily PM_10_ standard was retained without replacement by a PM_2.5–10_ standard because of lack of sufficient evidence of adverse health effects. The U.S. EPA will continue to monitor PM_2.5–10_ in sites throughout the United States and has recognized the need for further research on the health effects of coarse PM.

The National Research Council (2004) has outlined as a top research priority the identification of subpopulations at risk of adverse health outcomes related to PM exposure. Inner-city African-American children incur a disproportionate burden of asthma morbidity compared with other U.S. children (American Lung Association 2005; Moorman et al. 2007). Although there are many hypothesized contributors to the disparities in asthma morbidity in the United States, including access to medical care, exposure to child care settings or other children, diet, and stress, exposure to environmental pollutants may play an important role (Gold and Wright 2005). Inner-city African-American children with asthma may be a susceptible subpopulation because they are more likely to live in geographic areas with poor outdoor air quality (American Lung Association 2005), and ongoing exposure to home indoor air of suboptimal quality may confer additional risk to asthma health. The goal of the present study was to investigate the effect of in-home coarse and fine PM on respiratory symptoms, rescue medication use, and acute health care use among preschool asthmatic children living in inner-city Baltimore. We selected a population of inner-city, predominantly minority children as a potentially susceptible subpopulation.

## Materials and Methods

### Study design

The Baltimore Indoor Environmental Study of Asthma in Kids is a longitudinal study that was designed to investigate the role of indoor pollutants and allergens on asthma (Diette et al. 2007; Matsui et al. 2006). We evaluated participating children at baseline and at 3 and 6 months. At each time interval, environmental monitoring occurred for 3 consecutive days, and we assessed health outcomes through caregiver report. The Johns Hopkins Medical Institutional Review Board approved the study, and all participants provided informed consent before beginning the study.

### Participants

Recruitment for this study began in September 2001, and we completed the last follow-up visit in April 2004. We recruited participants from the health systems that provide care to most residents of East Baltimore, Maryland. To ascertain the diagnosis of asthma, we used a two-stage process. We identified children as potentially eligible if they had a health care encounter for asthma with an ICD-9 [*International Classification of Diseases, 9th Revision* (World Health Organization 1975)] code of 493.X in the previous 12 months. To confirm the diagnosis, participants had to report a physician diagnosis of asthma and had to have symptoms of asthma and/or medication use for asthma in the previous 6 months. Other inclusion criteria were age between 2 and 6 years and residence within one of nine contiguous ZIP codes within East Baltimore. Based on 2000 U.S. Census data, the ZIP codes of homes included in the study represented a geographic region that is > 99% urban (U.S. Census Bureau 2000).

### Air quality assessment

A trained environmental technician completed home visits. We conducted environmental monitoring at baseline and at 3 and 6 months. At each time period, we performed integrated air sampling in the child’s bedroom over a 3-day period. We chose the child’s bedroom as the indoor monitoring site because the bedroom represents an environment where we expected the child to spend a substantial portion of time while indoors. We conducted air sampling continuously over 72 hr using PM_10_ and PM_2.5_ 4 L/min MSP impactors (St. Paul, MN) loaded with 37-mm, 2.0-μm pore-size, Teflo polytetrafluoroethylene membrane filters with polypropylene support rings (Pall Corporation, Ann Arbor, MI). We calculated coarse PM fraction as the difference between PM_10_ and PM_2.5_, a method that has been commonly used to assess coarse PM concentrations (Brunekreef and Forsberg 2005; Vanderpool et al. 2004). Inlet flow rates were calibrated at the beginning and end of each sampling period using primary standards (DryCal; Bios International Corporation, Butler, NJ). PM gravimetric analysis was conducted on a Mettler T5 microbalance (Mettler Toledo, Inc., Columbus, OH), after filters were pre-equilibrated for 24 hr at constant temperature and humidity. We measured in-home temperature and humidity concurrently using a HOBO temperature and humidity data logger (Onset Corporation, Pocasset, MA). We measured ambient PM for the study at a central site located within the study area using standard methods (U.S. EPA 1997). All homes were within 2 miles of the central monitoring site. PM_2.5_ was collected using the PM_2.5_ Partisol-Plus model 2025 FRM sequential air sampler (Rupprecht & Patashnick Co. Inc., Albany, NY) and PM_10_ using a tapered element oscillating microbalance (TEOM 1400; Rupprecht & Patashnick). For this analysis, when ambient values were missing from the central site, values were supplemented from the Maryland Department of the Environment Old Town Station, a reporting site for the U.S. EPA ambient air monitoring network that is within 1 mile of the central monitoring site (U.S. EPA 2006a).

### Clinical evaluation

We evaluated participants at baseline and at 3 and 6 months. During the baseline clinic visit, each child underwent skin prick testing (Multi-Test II; Lincoln Diagnostics, Decatur, IL) to 14 aero-allergens: American and German cockroach, dust mite mix, cat dander, dog hair/dander, mouse epithelia, rat epithelia, three pollens (Eastern Oak mix, grass mix, ragweed mix), and four molds (*Helminthosporium*, *Alternaria*, *Penicillium*, and *Aspergillus*). Atopy was defined as at least one positive skin test result to the panel of allergens tested.

Once during each of the three monitoring periods, caregivers completed a health questionnaire that included closed-ended questions from the International Study of Asthma and Allergies in Childhood (Asher et al. 1995) and the Children’s Health Survey for Asthma (Asmussen et al. 1999) to evaluate indicators of asthma health. Questions ascertained information about acute health care use in the prior 3 months (emergency department visits, unscheduled doctor visits, and hospitalizations) and days of rescue medication use in the previous 2 weeks (short-acting beta agonist). We also asked caregivers about the child’s symptoms in the previous 2 weeks, including *a*) wheezing, coughing, or tightness in the chest; *b*) the need to slow down or stop activities because of asthma symptoms; *c*) wheezing so badly that the child could only speak one or two words at a time between breaths; *d*) symptoms with exercise; and *e*) nocturnal symptoms. We quantified each symptom as the number of days that the symptom was present in the previous 2 weeks (0–14 days).

Caregivers also completed a daily activity diary that detailed activities that occurred in the home during three time periods, morning (6000–1200 hours), afternoon (1200–1800 hours), and evening (1800–0600 hours). They recorded the number of windows that were open for > 10 min during each of these time periods. Caregivers also recorded the time that the child spent in the home, including the room where the environmental monitors were placed, in the daily diary.

### Statistical analysis

We generated summary statistics using means or proportions, as appropriate. We made comparisons using the chi-square test for proportions and Student’s *t*-test for continuous data. We used negative binomial regression models with generalized estimating equations (GEE) (Zeger and Liang 1986) to model the relationship between 3-day average PM and three repeated measures of days of symptoms or rescue medication use during the previous 2 weeks. We used logistic regression models with GEE to model the relationship between PM concentration and odds of an acute health care encounter (hospitalization, emergency department visit, and unscheduled doctor visit). We constructed multivariate models to account for potential confounders that we identified based on a known relationship with asthma or with PM, or based on a statistically significant association with either, in bivariate analyses. These covariates included age, sex, race, parent education level, season, and ambient PM. The number of open windows in the home was not included in the final multivariate models because the addition of this variable had no substantial effect on the results. We performed all analyses with StataSE statistical software (version 8.0; StataCorp, College Station, TX). Statistical significance was defined as *p* < 0.05.

## Results

The 150 children were between 2 and 6 years of age and were predominantly African American (91%) and from households of low socioeconomic status (Table 1). More than two-thirds of the children were atopic. The children had evidence of active asthma symptoms (Table 2), and 62% met classification criteria for persistent asthma (National Heart, Lung, and Blood Institute 1997, 2002). Children spent, on average, 14 of every 24 hr in their homes, and approximately half of this time they spent in the room where the environmental monitoring occurred.

### Characteristics of the PM measured indoors

**Figure 1:**
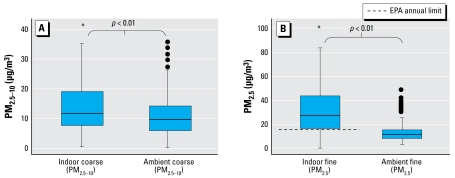
Indoor and ambient concentrations of PM_2.5–10_ (*A*) and PM_2.5_ (*B*). Boxes show the interquartile range (IQR), and the heavy dark lines are the median values. Whiskers represent the closest value within 1.5 times the IQR. Indoor PM concentrations were significantly higher than simultaneously measured ambient concentrations. The dashed line (*B*) indicates the U.S. EPA annual limit for ambient PM_2.5_. Eighty-five percent of homes had indoor PM_2.5_ concentrations that exceeded this ambient limit. There are currently no standards for ambient coarse PM. Asterisks indicate positive outliers, with values up to 288 μg/m^3^ for indoor PM_2.5–10_ (*A; n* = 24) and up to 216 μg/m^3^ for indoor PM_2.5_ (*B; n* = 27).

The mean (± SD) concentration for indoor PM_2.5–10_ was 17.4 ± 21.1 μg/m^3^, and for PM_2.5_ was 40.3 ± 35.4 μg/m^3^. The mean indoor PM_2.5_ concentrations did not differ significantly by season (summer, 37.6 ± 31.6 μg/m^3^; fall, 40.2 ± 33.3 μg/m^3^; winter, 40.2 ± 39.7 μg/m^3^; spring, 42.7 ± 36.6 μg/m^3^; *p* = 0.65). The mean indoor PM_2.5–10_ concentrations were significantly lower in the summer (12.83 ± 14.90 μg/m^3^) compared with the other seasons (fall, 18.14 ± 18.22 μg/m^3^; winter, 16.16 ± 12.19 μg/m^3^; spring, 20.85 ± 30.91 μg/m^3^; *p* < 0.01). The in-home PM_2.5–10_ and PM_2.5_ concentrations were significantly higher than the respective average ambient measurements made over the same time period (10.3 ± 21.0 μg/m^3^ and 12.4 ± 6.2; *p* < 0.01 for both comparisons) (Figure 1).

### Effects of coarse PM on asthma health

Higher concentrations of coarse PM measured indoors were associated with substantial increases in asthma symptoms in both the bivariate and the multivariate models (Tables 3, 4). For example, for every 10-ug/m^3^ increase in indoor PM_2.5–10_ concentration, there was a 6% [95% confidence interval (CI), 1 to 12%; *p* = 0.02] increase in the number of days of cough, wheeze, or chest tightness, after adjusting for age, race, sex, socioeconomic status, season, indoor fine PM, and ambient fine and coarse PM concentrations. In the adjusted models, higher indoor coarse PM concentration was also significantly associated with symptoms severe enough to slow a child’s activity, with wheezing that limited speaking ability, and with nocturnal symptoms with an 8% (95% CI, 2 to 14%; *p* = 0.01), an 11% (95% CI, 3 to 19%; *p* < 0.01), and an 8% (95% CI, 1 to 14; *p* = 0.02) increase in symptom days per 10-ug/m^3^ increase in PM_2.5–10_, respectively. For every 10-ug/m^3^ increase in PM_2.5–10_, there was a 6% (95% CI, 1 to 10%; *p* = 0.02) increase in days of rescue medication use, after adjusting for potential confounders. We found no significant associations between PM_2.5–10_ measured indoors and acute health care use, as measured by emergency department visits, unscheduled doctor visits, and hospitalizations (data not shown). Ambient PM_2.5–10_ was not significantly associated with respiratory symptoms, rescue medication use, or acute health care use in multivariate analyses adjusting for simultaneously measured indoor PM (data not shown).

### Effects of fine PM on asthma health

Fine PM was also positively associated with respiratory symptoms and with rescue medication use (Tables 3 and 4). For example, for every 10-μg/m^3^ increase in PM_2.5_ measured indoors, there was a 7% (95% CI, 0 to 14%; *p* = 0.04) increase in days of wheezing severe enough to limit speech and a 4% (95% CI, 1 to 8%; *p* = 0.04) increase in days on which rescue medication was needed, after adjustment for potential confounders. We found no significant associations between in-home or ambient PM_2.5_ concentrations and acute health care use (data not shown).

### Effects of PM on exercise-related symptoms

Both indoor and ambient fine PM concentrations were associated with exercise-related respiratory symptoms. In the bivariate models, for every 10-μg/m^3^ increase in indoor and ambient PM_2.5_, there was a 4% (95% CI, 1 to 7%; *p* = 0.04) and a 15% (95% CI, –4 to 35%; *p* = 0.13) increase in days of exercise-related symptoms, respectively. In the multivariate models adjusting for participant characteristics that were potential confounders as well as for simultaneous indoor and ambient coarse PM, for every 10-μg/m^3^ increase in indoor and ambient PM_2.5_, there was a 7% (95% CI, 2 to 11%; *p* < 0.01) and a 26% (95% CI, 1 to 52%; *p* = 0.04) increase in days of exercise-related symptoms, respectively. In contrast, neither indoor nor ambient coarse PM concentrations were associated with exercise-related symptoms.

## Discussion

Among inner-city children, most of whom were African American, we found that PM concentrations measured indoors were significantly associated with asthma symptoms and rescue medication use. In our cohort, indoor fine and coarse PM concentrations were associated with increases in respiratory symptoms that were clinically significant in terms of their magnitude. For example, a 10-μg/m^3^ increase in PM_2.5–10_ concentration was associated with an 8% increased incidence in days of symptoms severe enough to slow a child’s activity.

Our findings are consistent with what is known about the effects of indoor PM on childhood asthma and provide new evidence of a detrimental health effect of indoor coarse PM. In a previous study, Delfino et al. (2004) investigated PM exposures among 19 school-age children with asthma living in California and found that forced expiratory volume in 1 sec was inversely associated with personal, indoor, and ambient PM_2.5_ and PM_10_. They found stronger associations with indoor than with ambient PM concentrations among these children but did not evaluate the effects of PM on symptoms or medication use. Studies of school-age children in Seattle, Washington, have shown that indoor PM_2.5_ exposure was associated with decreased pulmonary function in a subgroup of 11 children not taking inhaled corticosteroids, but these studies did not include indoor measurements of PM_2.5–10_ (Koenig et al. 2005; Trenga et al. 2006). Thus, the present study, which includes a much larger sample size compared with previous studies, adds to the growing evidence that indoor PM exposure adversely affects asthma health and provides for the first time evidence that exposure to the indoor coarse (PM_2.5–10_) fraction is harmful for children with asthma, especially those of preschool age.

Our findings demonstrate that both indoor coarse and fine PM distinctly affect respiratory health. There are physiologic reasons that can explain why PM of these different size fractions can contribute separately to asthma morbidity. *In vitro* studies have shown that coarse PM preferentially induces inflammatory mediators in bronchial epithelial cells and alveolar macrophages compared with fine PM and that bacterial and endotoxin components of coarse PM may play a key role in this process (Becker et al. 2003, 2005). Although fine PM may be capable of reaching the alveoli, regions responsible for gas exchange, the deposition of coarse PM in conducting airways and subsequent bronchial hyperreactivity may be driving the symptomatic response measured in these preschool children. Although analyzing the composition of the PM and investigating the mechanism by which PM exacerbates asthma are beyond the scope of the present investigation, fine and coarse PM are known to have different sources, compositions, and deposition patterns in the respiratory tract, all of which may contribute to the differential health effects reported in our study. Understanding the components of indoor PM and the mechanism by which PM exacerbates asthma will be an important focus of future studies.

The strong relationship between indoor and ambient fine PM exposure and exercise-related symptoms was striking. Previous investigators have suggested that exercise may play a role in asthma by modifying the effect of environmental stimuli and pollutants. In a study of asthmatic children, McConnell et al. (2003) found that nitrogen dioxide was associated with bronchitic symptoms only among children participating in team sports. The authors hypothesized that the increased minute ventilation may in part explain why the effect of NO_2_ was modified by exercise. Our findings of increased exercise symptoms in response to fine PM exposure may be attributable to increased minute ventilation and an increased dose of fine PM in the distal airways and the pulmonary circulation that is more potent in eliciting exercise-related symptoms than the doses of coarse PM that deposit in the more proximal airways.

The study population is a key strength of our study design. We were able to enroll a study population of predominantly African-American, inner-city children who spent a substantial portion of time in their homes. Our results may provide insight about a potentially important contributor to the high burden of asthma in this population. African Americans are more likely than whites to live in geographic regions with poor outdoor air quality. For example, in 2002, 71% of African Americans lived in counties that violated federal air pollution standards, compared with 58% of the white population (American Lung Association 2005). Suboptimal indoor environments may contribute additional risk beyond that of the poor outdoor air quality. In Baltimore, a previous study has demonstrated that among children with asthma, those living in the inner city had in-home PM concentrations that were two to three times greater than did those in the local suburbs (Simons et al. 2007). Compared with what is considered acceptable quality for outdoor air, the children in the present study lived in homes with relatively elevated PM concentrations. For example, 85% of children lived in homes that would fail to meet the NAAQS annual standard for acceptable ambient air quality and 55% lived in homes that would fail to meet the more stringent 24-hr standard (U.S. EPA 1997). We have demonstrated that PM concentrations in the indoor air are associated with additional asthma morbidity after controlling for the effects of ambient PM measured simultaneously. Thus, indoor PM exposure may be an important contributor to the disproportionate burden of disease among African Americans living in inner cities.

Avoidance of harmful environmental exposures is a key component of national and international guideline recommendations for management of asthma (Global Initiative for Asthma 2006; National Heart, Lung, and Blood Institute 2007). Guidelines identify PM as a pollutant of concern, but specific recommendations are limited and focus mostly on avoiding exposure to elevated outdoor concentrations. This approach may imply that the indoor environment confers an advantage of lower exposure. However, our results demonstrate that the indoor environment may be less favorable in some circumstances. Although some previous studies have found indoor PM concentrations that are similar to or lower than outdoor concentrations (Koenig et al. 2005; Turpin et al. 2007), our findings are consistent with previous studies that have demonstrated indoor PM concentrations that greatly exceed outdoor concentrations (Breysse et al. 2005; Keeler et al. 2002; Wallace 1996; Wallace et al. 2003). Current recommendations for improving home indoor air quality focus mostly on avoidance of indoor environmental tobacco smoke. However, there are other important modifiable sources of indoor PM, including common cleaning and cooking activities (McCormack et al. 2008; Wallace et al. 2003). In urban environments especially, penetration of outdoor air, which contains traffic-related PM, into the indoors may also be an important contributor to the composition of the indoor air (Turpin et al. 2007). In the present study, the homes were all in the inner city, and most were close to the road, making exposure to traffic-related urban dust a likely contributing factor to the composition of PM in most homes. Thus, our results are most generalizable to populations of children who live in urban settings. Although our study cannot determine which sources of indoor PM are responsible for exacerbating asthma symptoms, previous studies have shown that PM concentrations can be lowered indoors (Eggleston et al. 2005). Such studies have shown that a multifaceted indoor environmental control approach can reduce asthma morbidity, but the independent effect of PM reduction has not yet been determined (Eggleston et al. 2005; Morgan et al. 2004). Intervention studies to assess the impact of improving indoor air quality on asthma symptoms and which PM reduction methods are most beneficial will be critical to provide data to inform guidelines and policy initiatives.

A limitation of this study is the potential for measurement error. Although the study area was relatively homogeneous, and all homes were within 2 miles of the central monitoring site, we took estimates of outdoor PM exposure from a central monitoring site rather than outdoor monitoring at the individual home. This approach could have resulted in nondifferential measurement error and may have contributed to the lack of a significant health effect of ambient PM exposure on non-exercise-related symptoms. The methods of sampling indoor, and ambient air differed and although we did not conduct side-by-side comparisons, systematic differences of up to 17% have been previously reported (Williams et al. 2000). We obtained the PM_2.5–10_ concentrations by subtracting PM _2.5_ concentrations from PM_10_, making the PM_2.5–10_ measurement subject to greater error than either individual measurement. We determined that the precision of the indoor PM_2.5_ and PM_10_ measures, determined by collocated samplers, were ±7% and ±6%, respectively, resulting in an expected error in PM_2.5–10_ of 9%, which is consistent with the precision described previously (Chen et al. 2007; Vanderpool et al. 2004; Williams et al. 2000). Importantly, we conducted environmental monitoring in the same manner for all study participants and did not differ it based on asthma status. Thus, nondifferential measurement error would be expected to bias our results toward the null and would not be responsible for the observed association between indoor PM concentrations and asthma symptoms in this study.

In the present longitudinal study, fine and coarse PM concentrations measured indoors were associated with increased asthma morbidity, including more frequent respiratory symptoms and rescue medication use, among inner-city minority children. Although the present study cannot delineate which sources of PM measured indoors were responsible for the observed health effects, these findings suggest that improving air quality in the indoor environment with a strategy that reduces PM concentration may improve asthma health. These findings do not negate the importance of optimizing outdoor air quality, because outdoor PM concentrations are linked to adverse health effects, especially among those with underlying pulmonary disease, and outdoor air is an important determinant of the composition of indoor air. Instead, improving indoor air quality and lowering indoor PM concentrations may provide an additional means of improving asthma health, especially among children living in inner cities.

## Figures and Tables

**Table 1:** Participant characteristics (*n* = 150).

Characteristic	Value
Age [years; mean (range)]	4.4 (2–6)
Race (%)	
African American	91
Caucasian	5
Other	4
Sex (% male)	58
Caregiver education level (%)	
Eighth grade/some high school	38
High school	43
Some college	19
Health insurance (%)	
Public	89
Private	9
Self-pay	2

**Table 2:** Baseline asthma status.

Characteristic	Value
Atopic (%)	69
Acute care use in the past 3 months (%)	
Emergency department visit	24
Hospitalization	3
Unscheduled doctor visit	18
Days of symptoms in the past 2 weeks (mean ± SD)	
Cough, wheezing, chest tightness	2.16 ± 3.37
Slow down because of symptoms	1.92 ± 3.79
Symptoms with running	1.60 ± 3.13
Nocturnal symptoms	1.69 ± 3.49
Limited speech	0.33 ± 1.20
Days of rescue medication use in the previous 2 weeks (mean ± SD)	3.77 ± 5.19

**Table 3:** Indoor PM concentrations, asthma symptoms, and rescue medication use: bivariate models.

	PM_2.5–10_ (per 10-μg/m^3^ increase)		PM_2.5_ (per 10-μg/m^3^ increase)	
Symptom	IRR (95% CI)	*p*-Value	IRR (95% CI)	*p*-Value
Cough, wheezing, chest tightness	1.05 (0.99–1.10)	0.08	1.01 (0.98–1.05)	0.41
Slow down	1.08 (1.03–1.13)	< 0.01	1.00 (0.97–1.04)	0.85
Symptoms with running	1.03 (0.97–1.09)	0.39	1.04 (1.01–1.07)	0.04
Nocturnal symptoms	1.06 (1.01–1.11)	0.03	1.02 (0.98–1.05)	0.37
Limited speech	1.11 (1.05–1.18)	< 0.01	1.01 (0.95–1.07)	0.33
Rescue medication use	1.06 (1.02–1.11)	< 0.01	1.03 (1.00–1.6)	0.06

IRR, incidence rate ratio.

**Table 4:** Indoor PM concentrations, asthma symptoms, and rescue medication use: multivariate models.

	PM_2.5–10_ (per 10 μg/m^3^ increase)^a^		PM_2.5_ (per 10 μg/m^3^ increase)^b^	
Symptom	IRR (95% CI)	*p*-Value	IRR (95% CI)	*p*-Value
Cough, wheezing, chest tightness	1.06 (1.01–1.12)	0.02	1.03 (0.99–1.07)	0.18
Slow down	1.08 (1.02–1.14)	0.01	1.04 (1.0–1.09)	0.06
Symptoms with running	1.00 (0.94–1.08)	0.81	1.07 (1.02–1.11)	< 0.01
Nocturnal symptoms	1.08 (1.01–1.14)	0.02	1.06 (1.01–1.10)	0.01
Limited speech	1.11 (1.03–1.19)	< 0.01	1.07 (1.00–1.14)	0.04
Rescue medication use	1.06 (1.01–1.10)	0.02	1.04 (1.01–1.08)	0.04

IRR, incidence rate ratio.

a Adjusted for age, sex, race, parent education level, season, indoor fine PM, ambient fine PM, ambient coarse PM.

b Adjusted for age, sex, race, parent education level, season, indoor coarse PM, ambient coarse PM, ambient fine PM.
